# Effect of Current Density on the Corrosion Resistance and Photocatalytic Properties of Cu-Ni-Zn_0.96_Ni_0.02_Cu_0.02_O Nanocomposite Coatings

**DOI:** 10.3390/ma16144925

**Published:** 2023-07-10

**Authors:** Haifeng Tan, Wenchao Yang, Mingzhu Hao, Chao Wang, Jie Yang, Haixuan Sunyu, Yunhe Ling, Guihong Song, Chunlin He

**Affiliations:** 1 Liaoning Provincial Key Laboratory of Advanced Materials, Shenyang University, Shenyang 110044, China; hftan8765@syu.edu.cn (H.T.);; 2 School of Material Science and Technology, Shenyang University of Technology, Shenyang 110870, China; ghsongsut@126.com

**Keywords:** (Cu, Ni) co-doped ZnO, Cu-Ni nanocomposite coating, electrodeposition, corrosion resistance, photocatalysis properties

## Abstract

2 at.% Cu + 2 at.% Ni were co-doped in ZnO nanoparticles by a simple hydrothermal method, and then the modified nanoparticles were compounded into Cu-Ni alloy coatings using an electroplating technique. The effects of the current density (15–45 mA/cm^2^) on the phase structure, surface morphology, thickness, microhardness, corrosion resistance, and photocatalytic properties of the coatings were investigated. The results show that the Cu-Ni-Zn_0.96_Ni_0.02_Cu_0.02_O nanocomposite coatings had the highest compactness and the best overall performance at a current density of 35 mA/cm^2^. At this point, the co-deposition rate reached its maximum, resulting in the deposition of more Zn_0.96_Ni_0.02_Cu_0.02_O nanoparticles in the coating. More nanoparticles were dispersed in the coating with a better particle strengthening effect, which resulted in a minimum crystallite size of 15.21 nm and a maximum microhardness of 558 HV. Moreover, the surface structure of the coatings became finer and denser. Therefore, the corrosion resistance was significantly improved with a corrosion current density of 2.21 × 10^–3^ mA/cm^2^, and the charge transfer resistance was up to 20.98 kΩ·cm^2^. The maximum decolorization rate of the rhodamine B solution was 24.08% under ultraviolet light irradiation for 5 h. The improvement in the comprehensive performance was mainly attributed to the greater concentration of Zn_0.96_Ni_0.02_Cu_0.02_O nanoparticles in the coating, which played the role of the particle-reinforced phase and reduced the microstructure defects.

## 1. Introduction

The service life and safety of marine infrastructures, such as ships, submarine pipelines, harbor terminals, and cross-sea bridges, are less than ideal, and corrosion often occurs, causing serious safety hazard problems, due to the harsh marine service environment. Most marine engineering structures are currently bare or underprotected, and reactive metals, such as aluminum, magnesium, iron, and alloys, are facing great challenges in serving the complicated marine environments [1,2,3,4].

Cu-Ni alloy parts and Cu-Ni alloy coatings have good resistance to seawater scouring corrosion, high heat transfer coefficients, excellent mechanical or welding properties, and can inhibit the adhesion of marine microorganisms. They are widely used in the cooling water pipes of the main and auxiliary engines of ships, fire-fighting pipelines of offshore oil recovery platforms, heat exchangers of power plants, and condensers of nuclear power plants [5,6]. However, the rapid development of the marine industry has put forward increasingly high requirements for materials used in marine engineering applications, and Cu-Ni alloy coatings still cannot satisfy the severe service demands of the complicated and varied marine environments.

The addition of Al_2_O_3_, ZrO_2_, TiN, Y_2_O_3_, graphene, and other nanoparticles can further improve the strength, hardness, wear resistance, and corrosion resistance of Cu-Ni coatings [7,8,9,10,11]. These conventional nanoparticle reinforcements tend to improve the seawater corrosion resistance of Cu-Ni alloy coatings but cannot further improve their marine microbial corrosion resistance. ZnO is a typical functional nanoparticle with high application potential in the fields of photocatalysts [12], antiseptics [13], and semiconductor devices [14,15]. Generally, the photocatalytic activity of nanoparticles can be effectively improved by doping [16,17]. Therefore, ZnO can also be doped with transition metal ions to enhance its photocatalytic activity [18,19,20]. Since the atomic radii of Cu and Ni are smaller than that of Zn, they can be used to replace the ZnO lattice to adjust the photocatalytic activity [21]. The results of several related studies have confirmed that Cu and Ni co-doped ZnO can significantly affect UV absorption and luminescence properties, thus stimulating photocatalytic activity [21,22,23,24]. Then, can Cu and Ni co-doped ZnO with photocatalytic activity be used as a nanoparticle reinforcement to further enhance the seawater and marine microbial corrosion resistance of Cu-Ni alloy coatings?

Cu and Ni co-doped ZnO nanoparticles can be synthesized by methods such as the hydrothermal method [22], electrochemical deposition [25], sol-gel [26], laser ablation [27], microwave-assisted synthesis [28], the spray pyrolysis technique [19], and co-precipitation [23]. A series of ZnO nanoparticles with different Cu and Ni doping levels were prepared by a hydrothermal method [29]. The results show that 2 at.% Cu + 2 at.% Ni co-doped ZnO nanoparticles were obviously superior to pure ZnO, single doped ZnO, and other amounts of (Cu, Ni) co-doped ZnO. Compared with coating preparation methods such as spraying, electroless plating, and laser melting, electroplating technology is simple, cost-effective, and industrially scalable [10,30]. In a previous study, 2 at.% Cu + 2 at.% Ni co-doped ZnO nanoparticles were effectively compounded with Cu-Ni coatings by an electroplating technique [31]. The effects of nanoparticle addition on the performance of the nanocomposite coatings were investigated, and the results showed that Cu and Ni co-doped ZnO nanoparticles could effectively improve the hardness, corrosion resistance, and photocatalytic degradation of organic pollutants of the Cu-Ni composite coatings. The photocatalytic activity can reflect its antibacterial and bactericidal ability from the side. Therefore, this study indirectly reflects the microbial corrosion resistance of the coatings by the photocatalytic degradation of organic pollutants. 

In general, the performance of electrodeposited coatings is mainly controlled by electrodeposition parameters, such as the bath temperature [32], current density (*J_k_*) [33,34], electrolyte composition [35], and electrolyte pH [36]. In this paper, the effects of current density on the corrosion resistance and photocatalytic properties of Cu-Ni-Zn_0.96_Ni_0.02_Cu_0.02_O nanocomposite coatings are further investigated to optimize the preparation process and enhance their comprehensive performance.

## 2. Experimental Materials and Methods

### 2.1. Synthesis of Zn_0.96_Ni_0.02_Cu_0.02_O Nanopowders

In the experiment, Zn_0.96_Ni_0.02_Cu_0.02_O nanoparticles were first prepared by a simple hydrothermal method. The precursor solutions were Zn(NO_3_)_2_∙6H_2_O, Ni(NO_3_)_2_∙6H_2_O, Cu(NO_3_)_2_∙3H_2_O, C_6_H_12_N_4_ (HMT), and we ensured that the molar ratio of Zn^2+^ to HMT in the precursor solution was 1:1. The surfactant was used with C_6_H_5_Na_3_O_7_∙2H_2_O in the solution. These experimental reagents are analytically pure and purchased from the China National Pharmaceutical Group Corporation (SINOPHARM). The doping amounts of Cu and Ni in ZnO nanoparticles were regulated by controlling the ratio of Cu(NO_3_)_2_∙3H_2_O to Ni(NO_3_)_2_∙6H_2_O. The details of the preparation procedure were reported in a previous study [31]. Finally, 2 at.% Cu and 2 at.% Ni were doped into ZnO nanoparticles, which were subsequently used as a nanoparticle reinforcements in the electrodeposited coatings.

### 2.2. Preparation of Cu-Ni-Zn_0.96_Ni_0.02_Cu_0.02_O Nanocomposite Coatings

Since the oxide film of aluminum is only one nanometer thick, it is prone to damage in the process of use, leading to corrosion of the aluminum alloy substrate. Therefore, in the experiment, the 2024 aluminum alloy sheets were selected as the cathode for electroplating, and 70–30 Cu-Ni alloy sheets were used as the anode. The Cu-Ni alloy coatings were expected to expand the application of aluminum alloy structural parts. The aluminum alloy substrate was subjected to mechanical polishing, alkaline washing, and acid washing before electroplating. A simple DC-regulated power supply was used for electroplating. The bath composition was 20 g/L CuSO_4_∙5H_2_O, 85 g/L NiSO_4_∙6H_2_O, 75 g/L C_6_H_5_O_7_Na_3_∙2H_2_O, and 0.2 g/L C_12_H_25_SO_4_Na, as shown in previous work [31]. The added amount of Zn_0.96_Ni_0.02_Cu_0.02_O nanoparticles was 4 g/L, the plating solution was neutral, the bath temperature was 45 °C, the electroplating time was 45 min, and the electromagnetic stirring rate was 300 rpm/min. The effects of the current density (15, 25, 35, 45 mA·cm^–2^) on the properties of the nanocomposite coatings were investigated.

### 2.3. Characterization Techniques

The crystal structure of the Cu-Ni-Zn_0.96_Ni_0.02_Cu_0.02_O nanocomposite coatings was analyzed by a TD-3500 X-ray diffractometer with Cu K*α* radiation (*λ* = 1.5406 Å). The diffraction angle 2*θ* ranged from 20° to 90° at a scanning speed of 12 °/min. A scanning electron microscope (S-4800, Hitachi, Tokyo, Japan) was used to characterize the surface morphology and cross-sectional thickness of the coatings with a test voltage of 5 kV. Energy disperse spectroscopy (EDS, Oxford, Abingdon, UK) was used to measure the compositions of Cu, Ni, and Zn in the nanocomposite coatings with a test voltage of 15 kV. The microhardness of the nanocomposite coatings was measured with a 402MVD digital Vickers hardness tester.

The effects of the current density on the corrosion resistance of the nanocomposite coatings were investigated using the polarization curves and electrochemical impedance spectroscopy (EIS) by a CHI 604E device. In the three-electrode cell, the Cu-Ni-Zn_0.96_Ni_0.02_Cu_0.02_O nanocomposite coatings were used as the working electrode. A saturated calomel electrode and a graphite electrode were used as the reference electrode and the counter electrode, respectively. The corrosion resistance measurements were performed in a corrosive medium of 3.5% NaCl solution, and EIS measurements were conducted with a sine wave amplitude of 10 mV and a frequency range of 10^5^ Hz to 10^–2^ Hz. The potential scanning rate was 0.166 mVs^–1^, and the potentiodynamic polarization curves were obtained from *E_ocp_* −500 m V to *E_ocp_* +800 m V. 

A UV-VIS spectrophotometer (Lambda 750S, Perkin-Elmer, Waltham, MA, USA) was used to test the photocatalytic degradation performance of the nanocomposite coatings against rhodamine B (RhB) solution under UV light irradiation. The prepared nanocomposite coatings were soaked in 100 mL of 8 mg/L RhB solution, and the reaction system was stirred thoroughly for 60 min in the dark to achieve an adsorption equilibrium. A 10 w UV lamp at room temperature was used for irradiation, and the degradation process was measured by a UV-VIS spectrophotometer at certain time intervals. The decolorization rate *η* of the RhB solution by the nanocomposite coatings was calculated according to the formula described in Ref [31].

## 3. Results 

### 3.1. Phase Structure

**Figure 1:**
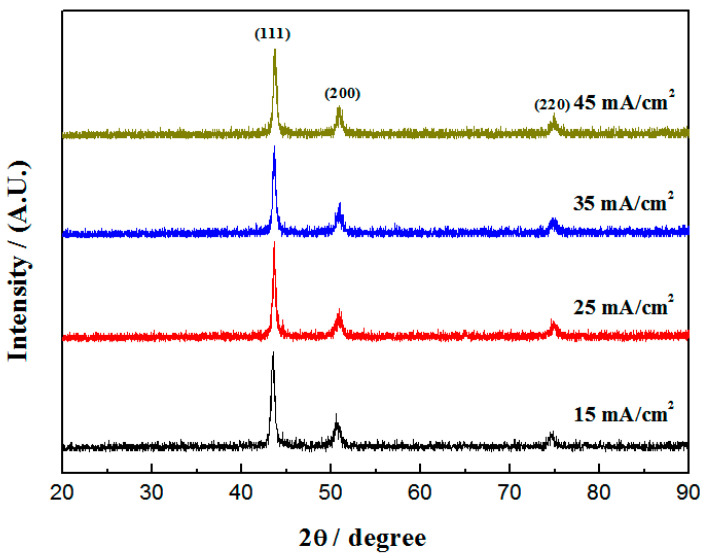
XRD patterns of Cu-Ni-Zn_0.96_Ni_0.02_Cu_0.02_O nanocomposite coatings with different current densities.

XRD was used to analyze the effect of the current density on the phase structure of the nanocomposite coatings, and the results are shown in Figure 1. Only the diffraction peaks of Cu-Ni solid solution were found, and no single diffraction peaks of Cu or Ni were found, which confirmed the successful preparation of the Cu-Ni alloy coatings [37,38]. The Cu-Ni-Zn_0.96_Ni_0.02_Cu_0.02_O nanocomposite coatings showed a dominant orientation of (111), (200), and (220) reflections, indicating good crystallization. With the increase in the current density, the diffraction angle of the (111) crystal plane gradually shifted toward higher values. This was due to the increase in the current density, which reduced the content of Cu atoms and increased the content of Ni atoms in the coatings, as shown in the following EDS results. Since the atomic radius of Ni is smaller than that of Cu, the lattice constant decreased, and the diffraction angle increased. The results showed that Cu and Ni co-doped ZnO nanoparticles showed typical hexagonal wurtzite, but the diffraction peaks of ZnO were not found in the XRD patterns of the nanocomposite coatings, probably because the content in the coatings was not enough to be detected [10,31].

The crystallite size of the Cu-Ni-Zn_0.96_Ni_0.02_Cu_0.02_O nanocomposite coatings was calculated from the (111) crystal plane diffraction peak of XRD, according to the Debye–Scherrer formula [39], and the results are shown in Table 1. By increasing the current density, the crystallite size in the coatings first decreased and then increased. The minimum crystallite size of 15.21 nm was obtained when the current density was 35 mA/cm^2^. Due to the increase in the current density, the cathodic electric field force increased, and the deposition rate increased. More Zn_0.96_Ni_0.02_Cu_0.02_O nanoparticles were deposited in the coatings, which hindered the grain growth [40]. Meanwhile, Zn_0.96_Ni_0.02_Cu_0.02_O could act as nucleation sites, causing the nucleation rate to increase and thus leading to a decrease in the crystallite size. 

According to Guglielmi’s two step model, the increasing incorporation rate can be attributed to the increasing tendency for ZnO nanoparticles to arrive at the cathode surface [41,42]. Therefore, it can be seen that the co-deposition rate was mainly determined by the nanoparticle content moving to the cathode surface and the current density. When the current density was too high, the nanoparticles at the far end could not move to the cathode surface in time, leading to a decrease in the content of nanoparticles in the coatings. Therefore, at a high current density, the deposition rate of Cu^2+^ and Ni^2+^ was higher than that of Zn_0.96_Ni_0.02_Cu_0.02_O nanoparticles, which led to a weaker grain refinement effect and an increase in the crystallite size of nanocomposite coatings. Overall, the effects of the current density on the crystallite size of the Cu-Ni coatings were not obvious, which is consistent with related reports [8,10,43].

### 3.2. Surface Morphology

**Figure 2:**
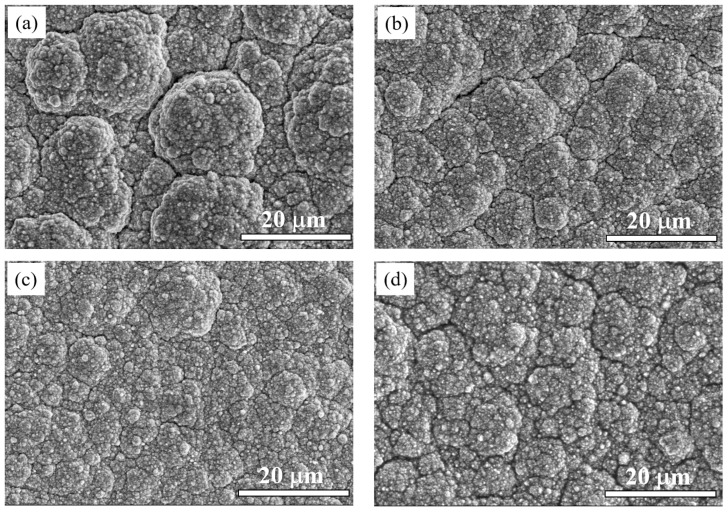
SEM images of Cu-Ni-Zn_0.96_Ni_0.02_Cu_0.02_O nanocomposite coatings with different current densities: (**a**) 15 mA/cm^2^, (**b**) 25 mA/cm^2^, (**c**) 35 mA/cm^2^, (**d**) 45 mA/cm^2^.

Figure 2 shows the SEM images of the surface morphology of the coatings with different current densities. Zn_0.96_Ni_0.02_Cu_0.02_O nanoparticles were irregular particles with uniform size and good dispersion, and the average particle size was about 60 nm [29]. At the current density of 15 mA/cm^2^, the coating surface showed some coarse cellular particles, a loose structure, more cracks and holes, and poor compactness. When the current density increased to 35 mA/cm^2^, the coating became uniform and dense, and the cracks were significantly reduced, as shown in Figure 2c. Sadoun, et al. had reported that nanoparticles could fill the microcracks and pores in the coatings, resulting in a dense and defect-less coatings [10,44]. When it continued to increase to 45 mA/cm^2^, more cracks appeared in the coating indicating that the quality of the coating began to deteriorate. 

At low current densities, the cathodic polarization was weak, the crystal growth rate was larger than the nucleation rate, and the coating structure was coarse. As the current density increased, cathodic polarization increased, the nucleation rate became faster, the grain size of the coating decreased, and the compactness became better. In the electrodeposition process, as nucleation sites, nanoparticles could form new grains and inhibit the continuous growth of grains, resulting in grain refinement [43]. However, when the current density was too high, the deposition rate of Cu and Ni ions was greater than the nucleation rate, and the nanoparticles at the far end could not move to the cathode surface in time. Therefore, the concentration of Zn_0.96_Ni_0.02_Cu_0.02_O nanoparticles in the coating would decrease and the grain refinement would be weakened, resulting in a slight increase in the grain size, which is consistent with the related reports [42,43].

### 3.3. Composition

**Figure 3:**
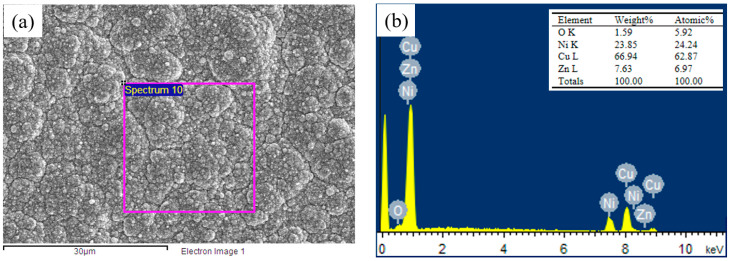
(**a**) SEM image and (**b**) EDS spectra of the nanocomposite coating at a current density of 35 mA/cm^2^.

**Figure 4:**
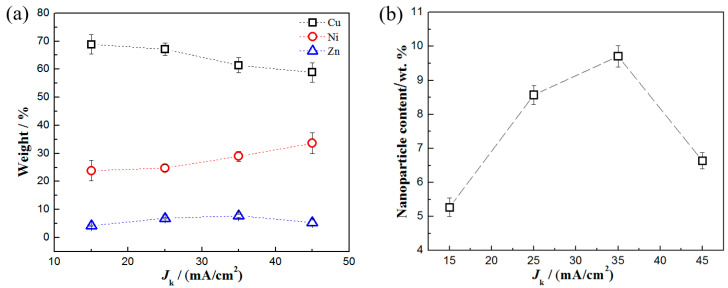
The contents of (**a**) Cu, Ni, Zn and (**b**) Zn_0.96_Ni_0.02_Cu_0.02_O nanoparticles in the coatings with different current densities.

The EDS elemental mapping analysis of the Cu-Ni-Zn_0.96_Ni_0.02_Cu_0.02_O nanocomposite coatings at a current density of 35 mA/cm^2^ is shown in Figure 3. The coating contained four main elements, Cu, Ni, Zn, and O, with a mass fraction of Cu that was approximately three times that of Ni. The atomic percentage of the Zn and O elements was close to 1:1, confirming that the co-doped ZnO nanoparticles were present in the coating. Figure 4a shows the EDS analysis of the Cu-Ni-Zn_0.96_Ni_0.02_Cu_0.02_O nanocomposite coatings at different current densities. After increasing the current density, the Cu content of the coatings decreased; on the contrary, the Ni content increased. This was because Cu^2+^ species were discharged by mass transport control, while Ni^2+^ species were discharged by activation control [45,46]. Hence, Cu was more easily deposited on the cathode surface at lower current densities, while with the increase in the current density, Ni was more likely to precipitate. 

The content of Zn increased first and then decreased with the increase in the current density, indicating that the content of Zn_0.96_Ni_0.02_Cu_0.02_O nanoparticles in the coatings increased first and then decreased. At a current density of 35 mA/cm^2^, the content of Zn in the coating reached the maximum value. According to the EDS results, the content of Zn_0.96_Ni_0.02_Cu_0.02_O nanoparticles in the coatings can be calculated as shown in Figure 4b. At a current density of 35 mA/cm^2^, the content of nanoparticles in the coating reached its maximum value. It has been reported that the content of ZrO_2_ [43] and Al_2_O_3_ [7] nanoparticles in Cu-Ni alloy coatings could be close to 10 wt.%, which is similar to the results of this work. The increase in the current density enhanced the cathode polarization and increased the co-deposition efficiency. Therefore, more nanoparticles were coated during the co-deposition process [38]. However, when the current density was too high, the deposition rate of metal ions was higher than that of nanoparticles, which would have led to a decrease in the content of nanoparticles in the coating. Hence, the content of nanoparticles directly affects the hardness, corrosion resistance, and photocatalytic performance of the coatings.

### 3.4. Thickness

**Figure 5:**
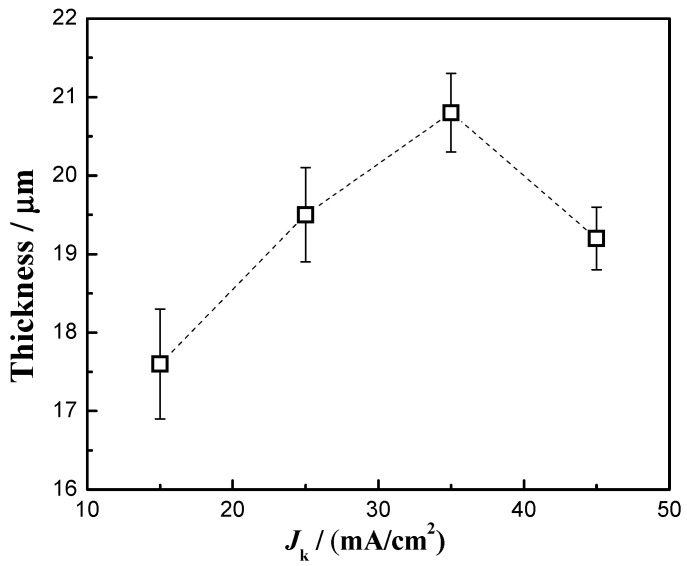
Thickness of Cu-Ni-Zn_0.96_Ni_0.02_Cu_0.02_O nanocomposite coatings at different current densities.

Figure 5 shows the thickness of the Cu-Ni-Zn_0.96_Ni_0.02_Cu_0.02_O nanocomposite coatings at different current densities. When the current density was 35 mA/cm^2^, the thickness of the Cu-Ni-Zn_0.96_Ni_0.02_Cu_0.02_O nanocomposite coating reached the maximum value. This was due to the weaker cathodic polarization and slower deposition rate at a current density of 15 mA/cm^2^, resulting in a thinner coating. When the current density was 35 mA/cm^2^, the cathode overpotential, electric field force, and the electroplating rate increased, resulting in an increase in the coating thickness. As the current density increased to 45 mA/cm^2^, the hydrogen evolution reaction intensified, the electroplating deposition rate slowed down instead [47], and the thickness of the Cu-Ni-Zn_0.96_Ni_0.02_Cu_0.02_O nanocomposite coating decreased. Moreover, nanoparticles serving as nucleation sites were beneficial for improving the efficiency of electrodeposition. Therefore, when the current density was 35 mA/cm^2^, the content of co-deposited nanoparticles in the coatings was the highest, ultimately achieving the maximum coating thickness.

### 3.5. Microhardness

**Figure 6:**
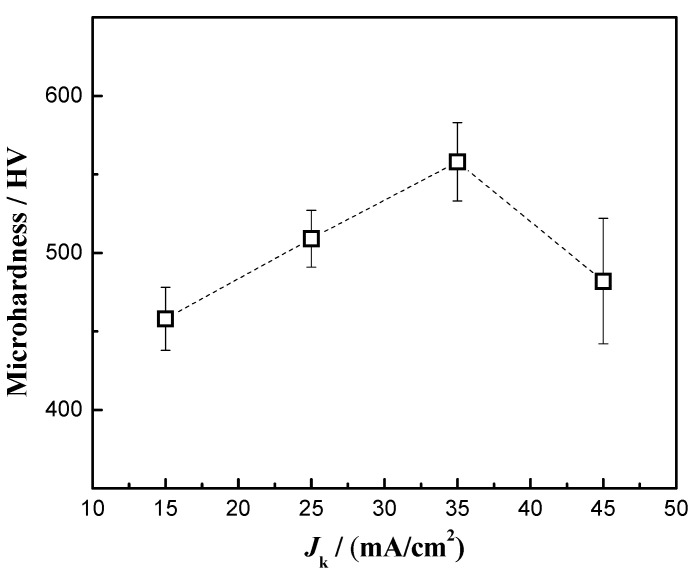
Microhardness of Cu-Ni-Zn_0.96_Ni_0.02_Cu_0.02_O nanocomposite coatings at different current densities.

Figure 6 shows the microhardness results of the nanocomposite coatings at different current densities. At a current density of 35 mA/cm^2^, the maximum microhardness of the nanocomposite coating was 558 HV. This hardness value was close to 600 HV, which is similar to the results reported recently [7,43]. As the current density increased from 15 mA/cm^2^ to 35 mA/cm^2^, the content of Zn_0.96_Ni_0.02_Cu_0.02_O nanoparticles in the coatings increased, and the surface of the coatings became smoother and denser. More nanoparticles and a better surface quality enabled the coatings to achieve maximum hardness. 

The strengthening mechanisms of Orowan looping and load transfer could play a major role in improving the hardness of the coatings [48,49]. The particles are less than 100 nm and dispersed uniformly in the coatings. When the indenter penetrate into the coatings, the Zn_0.96_Ni_0.02_Cu_0.02_O nanoparticles carry the load and impede the motion of dislocations [50]. The nanoparticles in the coatings hinder the deformation and improve the deformation resistance, thus increasing the hardness. A higher nanoparticle content, more uniform dispersion and fewer defects in the coatings lead to a higher hardness. At 45 mA/cm^2^, the Zn_0.96_Ni_0.02_Cu_0.02_O content of the nanocomposite coatings decreased, the crystallite size increased slightly, and the compactness worsened. The nanoparticle strengthening effect became weaker, and the microhardness of the coatings decreased. Therefore, the nanocomposite coatings obtained the highest hardness when the bath temperature was 45 °C and the current density was 35 mA/cm^2^.

### 3.6. Corrosion Resistance

**Figure 7:**
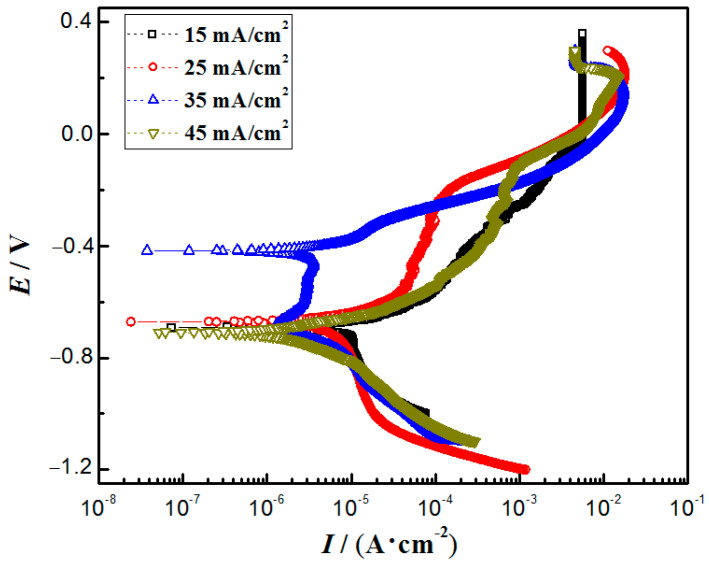
Polarization curves of Cu-Ni-Zn_0.96_Ni_0.02_Cu_0.02_O nanocomposite coatings at different current densities.

The polarization curves of the nanocomposite coatings at different current densities are shown in Figure 7, and Table 2 shows the Tafel fitting results of the polarization curves. The corrosion potential of the nanocomposite coatings varied little from 15 mA/cm^2^ to 25 mA/cm^2^. At a current density of 35 mA/cm^2^, the most positive corrosion potential of the coating was −0.43 V, and the lowest corrosion current density was 2.21 × 10^–3^ mA/cm^2^, indicating the slowest corrosion rate and the best corrosion resistance. When the bath temperature was 45 °C and the current density was 35 mA/cm^2^, the coating had the most nanoparticles and the densest structure, resulting in the best corrosion resistance [10]. In addition, the maximum coating thickness would also delay the corrosion process. The corrosion current density of the coatings in this work was similar to that of the coatings with ZrO_2_ [43] and Y_2_O_3_ [10], but slightly lower than that of the coatings with Al_2_O_3_ [7] and graphene [11].

**Figure 8:**
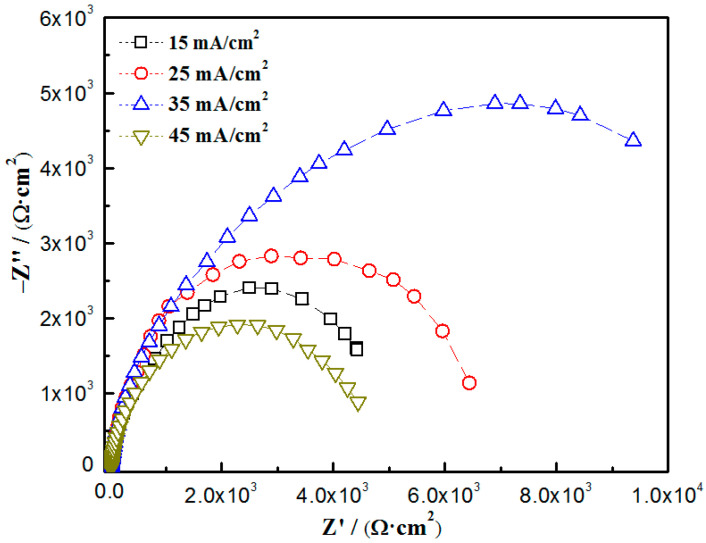
Nyquist diagrams of Cu-Ni-Zn_0.96_Ni_0.02_Cu_0.02_O nanocomposite coatings at different current densities.

**Figure 9:**
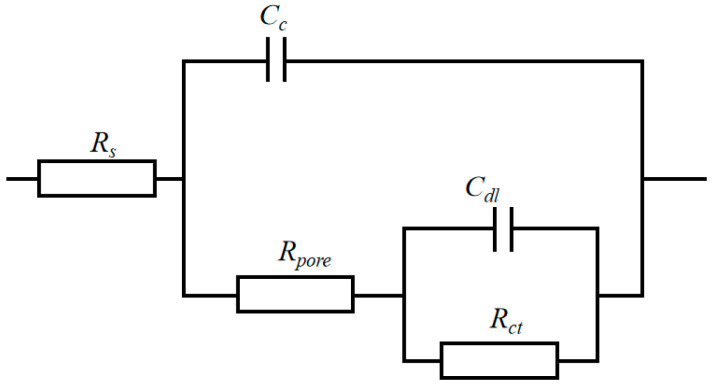
Equivalent circuit for fitting the EIS of Cu-Ni-Zn_0.96_Ni_0.02_Cu_0.02_O nanocomposite coatings.

Figure 8 shows the Nyquist plots of the nanocomposite coatings at different current densities. The equivalent electrical circuit of the nanocomposite coatings is shown in Figure 9, and the fitted results are shown in Table 3. *R_s_*, *R_pore_*, and *R_ct_* represent the solution resistance, micropore resistance, and charge transfer resistance, respectively. *C_c_* is the coating capacitance, whereas *C_dl_* is the electric double layer capacitance. At a current density of 35 mA/cm^2^, the nanocomposite coatings exhibited the largest capacitive arc resistance radius, indicating the best corrosion resistance. Meanwhile, *R_ct_* reached a maximum value of 20.978 kΩ·cm^2^, which was much higher than that of the coatings prepared at other current densities. The results were consistent with the polarization curves, indicating that the coatings prepared at a current density of 35 mA/cm^2^ had the best corrosion resistance.

The mechanical properties and corrosion resistance of the particle-reinforced nanocomposite coatings are mainly related to the properties of the nanoparticles, the defects in the coatings, the dispersion of the particles in the coatings, and the interfacial bonding between the particles and the metal matrix [48,51]. With the increase in the current density, the Zn_0.96_Ni_0.02_Cu_0.02_O concentration in the coatings gradually increased, which would be beneficial for filling the microvoids and microcracks in the coatings, resulting in a denser structure, thus improving the corrosion resistance. When the current density was too large, the deposition rate of the Cu and Ni ions was too high, the content of Zn_0.96_Ni_0.02_Cu_0.02_O nanoparticles in the coatings decreased, and defects such as cracks and holes in the nanocomposite coatings increased, resulting in a decrease in the corrosion resistance [7,52]. In a comprehensive comparison, the nanocomposite coatings reached the optimum corrosion resistance at a bath temperature of 45 °C and a current density of 35 mA/cm^2^.

### 3.7. Photocatalytic Performance

**Figure 10:**
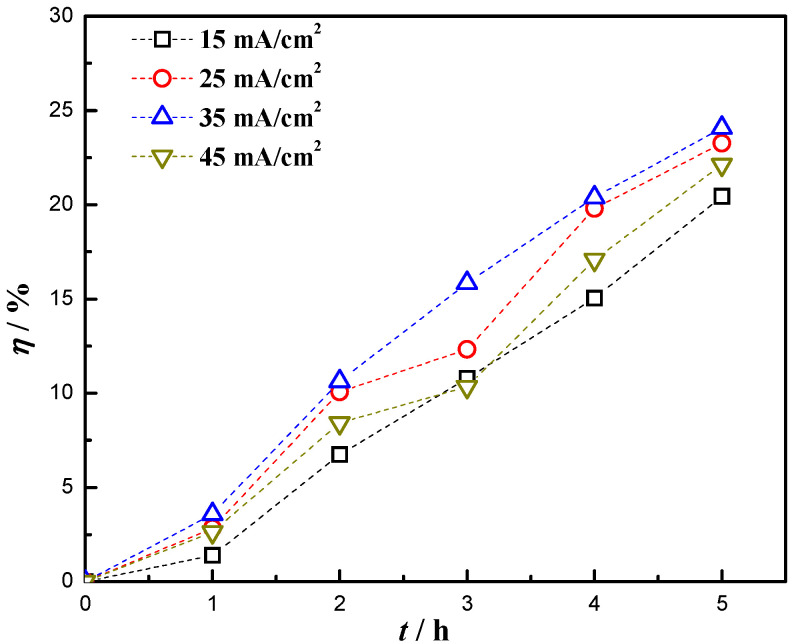
Illumination time and decolorization rate of the Cu-Ni-Zn_0.96_Ni_0.02_Cu_0.02_O nanocomposite coating at different current densities.

The optical band gap energy value of Zn_0.96_Ni_0.02_Cu_0.02_O nanoparticles could be reduced to 2.89 eV which was smaller than that of pure ZnO, exhibiting better photocatalytic activity [29]. According to the decolorization rate formula [31], the decolorization rate *η* of the RhB solution by nanocomposite coatings at different current densities was calculated, as shown in Figure 10. After 5 h of degradation with ultraviolet light, the *η* of RhB solution by Zn_0.96_Ni_0.02_Cu_0.02_O nanoparticles reached more than 90% [29]. When the current density increased from 15 mA/cm^2^ to 45 mA/cm^2^, the *η* values of RhB solution by nanocomposite coatings were 20.44%, 23.25%, 24.08%, and 22.12%, respectively, after 5 h of degradation, and the highest *η* was achieved at 35 mA/cm^2^. 

The *η* of RhB solution was related to the content of Zn_0.96_Ni_0.02_Cu_0.02_O nanoparticles in the coatings. At a current density of 35 mA/cm^2^, the content of nanoparticles in the coatings was the highest, so the nanocomposite coating was the most effective for the photocatalytic degradation of RhB solution. Due to the excellent bactericidal effect of ZnO nanoparticles [13] and the excellent photocatalytic activity exhibited by the doped nanoparticles, the addition of Zn_0.96_Ni_0.02_Cu_0.02_O nanoparticles not only improved the mechanical properties and seawater corrosion resistance of the nanocomposite coatings but also improved their marine microbial corrosion resistance. This provides a new idea for the development of functional nanoparticle reinforced metal matrix composites. 

## 4. Conclusions

The Cu-Ni-Zn_0.96_Ni_0.02_Cu_0.02_O nanocomposite coatings were prepared by a simple electrodeposition method. The effects of the current density on the phase structure, surface morphology, composition, thickness, hardness, corrosion resistance, and photocatalytic properties of the nanocomposite coatings were investigated.

(1) Diffraction peaks on the (111), (200), and (220) crystal planes were present in the nanocomposite coatings, and the coatings were well-crystallized. At a current density of 35 mA/cm^2^, the co-deposition rate reached its maximum, resulting in the deposition of more Zn_0.96_Ni_0.02_Cu_0.02_O nanoparticles in the coating. However, having more nanoparticles was beneficial for reducing defects such as microcracks and pores, ultimately increasing the compactness of the coating.

(2) Having more Zn_0.96_Ni_0.02_Cu_0.02_O nanoparticles uniformly dispersed in the coatings could effectively hinder the movement of dislocations, thereby increasing its hardness up to 558 HV. The lowest current density of 2.21 × 10^–3^ mA/cm^2^ and the highest charge transfer resistance of 20.98 kΩ·cm^2^ indicated that the coating had good corrosion resistance, which was attributed to its dense and defectless coating structure. 

(3) At the current density of 35 mA/cm^2^, the highest decolorization rate of RhB solution was 24.08% after 5 h of ultraviolet light exposure, due to the higher concentration of Zn_0.96_Ni_0.02_Cu_0.02_O nanoparticles in the coatings. The addition of Zn_0.96_Ni_0.02_Cu_0.02_O nanoparticles made the coating exhibit good photocatalytic activity, which would be beneficial for improving the marine microbial corrosion resistance of the Cu-Ni alloy coatings.

## Figures and Tables

**Table 1:** Diffraction angle of the (111) crystal plane and the crystallite size of the coatings at different current densities.

Current Densities/(mA/cm^2^)	(111) Crystal Plane Diffraction Angle/°	Crystallite Size/nm
15	43.51	16.13
25	43.64	15.65
35	43.72	15.21
45	43.77	15.53

**Table 2:** Tafel fitting results of polarization curves of Cu-Ni-Zn_0.96_Ni_0.02_Cu_0.02_O coatings with different current densities.

Current Densities/(mA/cm^2^)	*I_corr_*/(mA/cm^2^)	*E_corr_*/V	*E_pit_*/V
15	8.03 × 10^–3^	−0.67	−0.25
25	7.13 × 10^–3^	−0.66	−0.20
35	2.21 × 10^–3^	−0.43	−0.15
45	4.14 × 10^–3^	−0.68	−0.13

**Table 3:** Fitting results of the equivalent circuit of Cu-Ni-Zn_0.96_Ni_0.02_Cu_0.02_O nanocomposite coatings at different current densities.

Current Densities/(mA/cm^2^)	*R_s_* /(Ω·cm^2^)	*C_c_* /(S·cm^−2^·s^−n^)	n	*R_pore_* /(kΩ·cm^2^)	*C_dl_* /(S·cm^−2^·s^−n^)	n	*R_ct_* /(kΩ·cm^2^)
15	11.27	1.54 × 10^−4^	0.91	0.33	5.38 × 10^−4^	0.65	4.90
25	15.77	6.80 × 10^−4^	0.92	2.92	4.18 × 10^−4^	0.90	6.51
35	19.58	4.11 × 10^−5^	0.91	5.92	1.86 × 10^−8^	0.47	20.98
45	15.67	1.04 × 10^−4^	0.90	0.67	5.01 × 10^−4^	0.99	4.47

## Data Availability

Not applicable.
